# *Chaetopteryx bucari* sp. n., a new species from the *Chaetopteryx rugulosa* group from Croatia (Insecta, Trichoptera, Limnephilidae) with molecular, taxonomic and ecological notes on the group

**DOI:** 10.3897/zookeys.320.4565

**Published:** 2013-07-31

**Authors:** Mladen Kučinić, Ildikó Szivák, Steffen U. Pauls, Miklós Bálint, Antun Delić, Ivan Vučković

**Affiliations:** 1 Department of Biology (Group for Systematic Zoology & Entomology), Faculty of Science, University of Zagreb, Rooseveltov trg 6, 10000 Zagreb, Croatia; 2 Balaton Limnological Institute, Centre for Ecological Research, Hungarian Academy of Science, H-8237 Tihany, Klebelsberg Kuno u. 3, Hungary; 3 Biodiversity and Climate Research Centre (BiK-F), Senckenberg Gesellschaft für Naturforschung, Senckenberganlage 25, 60325 Frankfurt am Main, Germany; 4 Faculty of Ecudation, Department in Petrinja, University of Zagreb, Matice Hrvatske 12, 44250, Petrinja, Croatia; 5 Elektroprojekt d.d., Civil and Architectural Engineering Department, Alexandera von Humboldta 4, 10000 Zagreb, Croatia

**Keywords:** *Chaetopteryx*, aquatic insects, new species, distribution, Croatia

## Abstract

We describe a new autumnal caddisfly species *Chaetopteryx bucari* **sp. n.** from 8 localities in the Banovina region of Croatia. We also present molecular, taxonomic and ecological notes (emergence, sex ratio and seasonal dynamics) on the new species and discuss the distribution of *Chaetopteryx* species in general and the *Chaetopteryx rugulosa* group in particular. Based on Bayesian phylogenetic analysis *Chaetopteryx rugulosa schmidi* was separated from the clade containing the other subspecies of *Chaetopteryx rugulosa*. Thus the subspecies *Chaetopteryx rugulosa schmidi* is here raised to species level, *Chaetopteryx schmidi*, as it was described originally. We further present distribution data on rare species in the genus *Chaetopteryx* in Croatia.

## Introduction

The genus *Chaetopteryx* belongs to a small number of caddisfly genera with adults that are adapted to low air temperatures and emerge in autumn or winter, mostly from October-January. The larvae of most species live in small headwater streams and springs. This genus is distributed in Europe and parts of Asia (e.g., Asia Minor, Iran) (Malicky 2004, Lodovici and Valle 2007, Sipahiler 2010). In Europe, *Chaetopteryx* comprises 25 species (Malicky 2004, Lodovici and Valle 2007, Oláh 2011a, 2011b). A particularly interesting species group in the genus is the *Chaetopteryx rugulosa* group. This radiation consists of 6 species and 3 subspecies: *Chaetopteryx rugulosa rugulosa* Kolenati, 1848; *Chaetopteryx rugulosa mecsekensis* Nógrádi, 1986; *Chaetopteryx rugulosa noricum* Malicky, 1976; *Chaetopteryx rugulosa schmidi* Botosaneanu, 1957; *Chaetopteryx clara* McLachlan, 1876; *Chaetopteryx euganea* Moretti & Malicky, 1986; *Chaetopteryx goricensis* Malicky & Krušnik, 1986; *Chaetopteryx irenae* Krušnik & Malicky, 1986 and *Chaetopteryx marinkovicae* Malicky and Krušnik, 1988 (Malicky 2004).

Four years ago we started systematically collecting adults of the genus *Chaetopteryx*, including members of the *Chaetopteryx rugulosa* group in Croatia. This paper has 2 main objectives, first to present and describe a new species from the *Chaetopteryx rugulosa* group found in Croatia, and second to present new molecular, taxonomic, distributional, and ecological information on the *Chaetopteryx rugulosa* group.

## Material and methods

**Figure 1.**
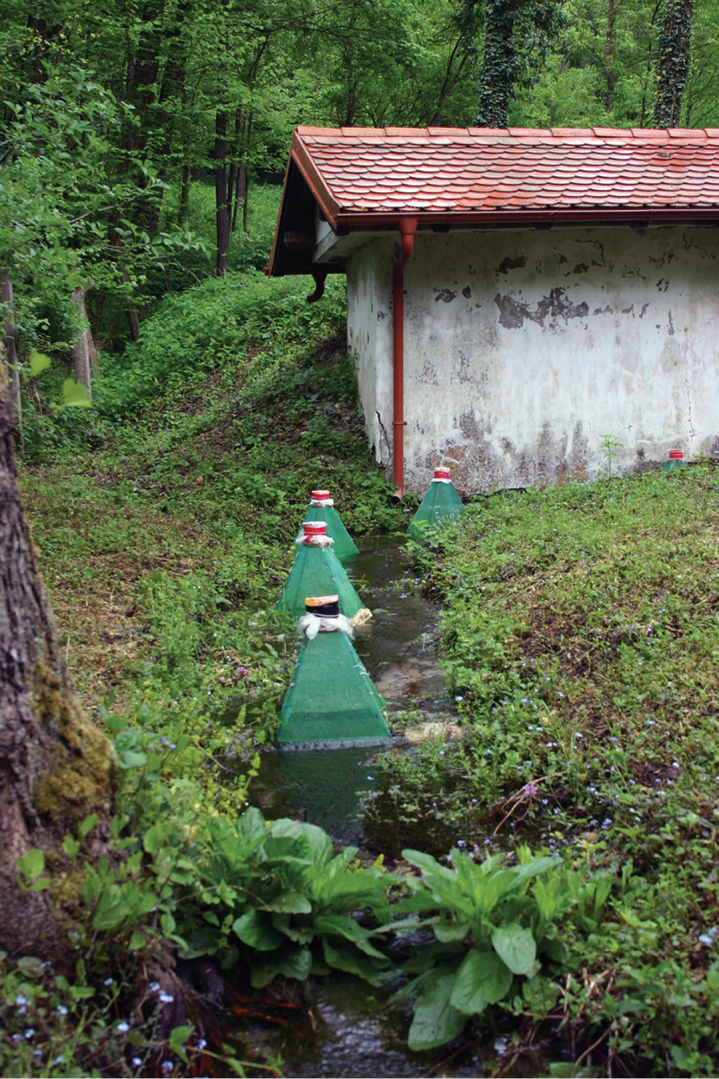
Type locality of *Chaetopteryx bucari* sp. n., showing pyramid-type emergence traps, Pecki spring, Croatia.

**Fieldwork.** We collected specimens of *Chaetopteryx* including *Chaetopteryx rugulosa* group species in the continental (central Croatia, Banovina, Hrvatsko zagorje, Kordun, Slavonia), mountain (Gorski kotar, Lika regions) and Mediterranean (Istria and Dalmatia) regions of Croatia. Collecting methods included the use of entomological nets and handpicking specimens from walls of small buildings or wells, or from the riparian vegetation near springs and headwater streams. In one spring (Pecki spring, Banovina region) (Table 1) we installed 5 pyramid-type emergence traps in 2010 and 2011 to investigate the emergence dynamics of caddisflies (Figure 1). This investigation is part of a multi-year study on emergence dynamics of aquatic insects in springs and other aquatic habitats in Croatia and the Dinaric karst of the Balkan Peninsula (Bosnia and Herzegovina) (Kučinić 2002, Previšić et al. 2007, Ivković et al. 2011, Semnički et al. 2011, 2012, M. Kučinić unpublished data). The emergence trapping methodology was presented in detail by Kučinić (2002) and Previšić et al. (2007).

**Table 1.** Localities where *Chaetopteryx bucari* sp. n., was collected, including habitat type, elevation (m a.s.l.), and geographic coordinates.

**Location**	**Character of location**	**Altitude (m)**	**Geographic coordinates**
Bijele stijene	wellspring and stream	144	45°25'23"N, 16°13'23"E
Gore	wellspring	165	45°24'21"N, 16°14'22"E
Hrvatski Čuntić	stream	159	45°21'28"N, 16°17'04"E
Marića točak	wellspring	163	45°21'29"N, 16°17'03"E
Pašino vrelo	spring	185	45°17'16"N, 16°25'13"E
Pecki	spring	161	45°23'50"N, 16°14'40"E
Slabinja	wellspring	104	45°13'05"N, 16°37'52"E
Varoški bunar	wellspring	130	45°13'34"N, 16°33'12"E

In pyramid-type emergence traps caddisflies were collected in 1% formaldehyde and thereafter stored in 80% alcohol. All other collected specimens were stored directly in 80% or 96% alcohol. All specimens were deposited in the collections of the first and second authors. The holotype is deposited in the Croatian Natural History Museum in Zagreb.

**Laboratory work.** For the phylogenetic analysis we compiled mtCOI DNA sequence data for 103 specimens from the *Chaetopteryx rugulosa* group (Table 2). We also sequenced several outgroup taxa of varying putative phylogenetic depths including congeneric species (e.g., *Chaetopteryx gessneri* McLachlan, 1876, *Chaetopteryx fusca* Brauer, 1857, *Chaetopteryx major* McLachalan, 1876, *Chaetopteryx villosa* (Fabricius, 1798)), other members of the tribe Chaetopterygini (*Chaetopterygopsis maclachlani* (Stein, 1874)), other members of the subfamily Limnephilinae (*Limnephilus centralis* Curtis, 1834), and members of a different subfamily of Limnephilidae (e.g. *Metanoea rhaetica* Schmid, 1955, *Drusus alpinus* (Meyer-Dür, 1875), *Drusus rectus* McLachlan, 1868).

Systematic presentation follows Morse (2013). The terminology and morphological assessment of the *Chaetopteryx rugulosa* group follows Malicky et al. (1986), Malicky and Krušnik (1988), Urbanič and Krušnik (2003), Botosaneanu and Giudicelli (2004), Holzenthal et al. (2007), Oláh (2011a), and Vučković et al. (2011). Comparative assessments of morphological features of *Chaetopteryx bucari* were based on the other specimens collected in Croatia (*Chaetopteryx rugulosa rugulosa*, *Chaetopteryx marinkovicae*) or based on literature (e.g., *Chaetopteryx rugulosa schmidi*, *Chaetopteryx rugulosa mecsekensis*, Malicky et al. 1986, Malicky 2004). Morphological features of genitalia of *Chaetopteryx bucari* were analysed from 84 specimens (40 males and 44 females).

**Table 2.** List of species included in the DNA analysis (mtCOI sequences). Localities are given with country code, locality/specimen data, and collection date.

**Species name**	**Locality**	**Specimen ID**	**Accession number**	**Collectors/Source**
*Chaetopteryx aproka*	ROU, Ignis Mts., springs near Desesti-Statiunea Izvoare, 21.10.2010	CAxJC0101	HE858253	Ecsedi, Olah & Szivak
*Chaetopteryx aproka*	ROU, Ignis Mts., springs near Desesti-Statiunea Izvoare, 21.10.2010	CAxJC0102	HE858254	Ecsedi, Olah & Szivak
*Chaetopteryx aproka*	ROU, Ignis Mts., springs near Desesti-Statiunea Izvoare, 21.10.2010	CAxJC0103	HE858255	Ecsedi, Olah & Szivak
*Chaetopteryx bosniaca*	BIH, Livno, Sturba river, 08.11.2009	CBxED0101		Kučinić, Delić & Mihoci
*Chaetopteryx bosniaca*	BIH, Livno, Sturba river, 08.11.2009	CBxED0102		Kučinić, Delić & Mihoci
*Chaetopteryx bosniaca*	BIH, Livno, Sturba river, 08.11.2009	CBxED0103		Kučinić, Delić & Mihoci
*Chaetopteryx bosniaca*	BIH, Livno, Sturba river, 08.11.2009	CBxED0104		Kučinić, Delić & Mihoci
*Chaetopteryx bosniaca*	BIH, Livno, Sturba river, 08.11.2009	CBxED0105		Kučinić, Delić & Mihoci
*Chaetopteryx clara*	SLO, Ljubljana, Mostec park, Przanec stream, 06.12.2009	CCxEA0101	JF891164	Dery & Szivak
*Chaetopteryx clara*	SLO, Ljubljana, Mostec park, Przanec stream, 06.12.2009	CCxEA0102	JF891165	Dery & Szivak
*Chaetopteryx clara*	SLO, Ljubljana, Mostec park, Przanec stream, 06.12.2009	CCxEA0103	JF891166	Dery & Szivak
*Chaetopteryx clara*	SLO, Ljubljana, Mostec park, Przanec stream, 06.12.2009	CCxEA0104	JF891167	Dery & Szivak
*Chaetopteryx clara*	SLO, Ljubljana, Mostec park, Przanec stream, 06.12.2009	CCxEA0105	JF891168	Dery & Szivak
*Chaetopteryx goricensis*	SLO, spring of Lokavscek stream near Predmeja, 06.12.2009	CGREG0101	JF891159	Dery & Szivak
*Chaetopteryx goricensis*	SLO, spring of Lokavscek stream near Predmeja, 06.12.2009	CGREG0102	JF891160	Dery & Szivak
*Chaetopteryx goricensis*	SLO, spring of Lokavscek stream near Predmeja, 06.12.2009	CGREG0103	JF891161	Dery & Szivak
*Chaetopteryx goricensis*	SLO, spring of Lokavscek stream near Predmeja, 06.12.2009	CGREG0104	JF891162	Dery & Szivak
*Chaetopteryx goricensis*	SLO, spring of Lokavscek stream near Predmeja, 06.12.2009	CGREG0105	JF891163	Dery & Szivak
*Chaetopteryx goricensis*	SLO, spring near Čekovnik (Hlevise), 05.12.2009	CGREG0201	JF891154	Dery & Szivak
*Chaetopteryx goricensis*	SLO, spring near Čekovnik (Blask), 05.12.2009	CGREG0301	JF891155	Dery & Szivak
*Chaetopteryx goricensis*	SLO, spring near Čekovnik (Blask), 05.12.2009	CGREG0302	JF891156	Dery & Szivak
*Chaetopteryx goricensis*	SLO, spring near Čekovnik (Blask), 05.12.2009	CGREG0303	JF891157	Dery & Szivak
*Chaetopteryx goricensis*	SLO, spring near Čekovnik (Blask), 05.12.2009	CGREG0304	JF891158	Dery & Szivak
*Chaetopteryx irenae*	SLO, Susica stream near Misliče, 06.12.2009	CIxEI0101	JF891169	Dery & Szivak
*Chaetopteryx irenae*	SLO, Susica stream near Misliče, 06.12.2009	CIxEI0102	JF891170	Dery & Szivak
*Chaetopteryx irenae*	SLO, Misliče, Susica stream, 06.12.2009	CIxEI0103	JF891171	Dery & Szivak
*Chaetopteryx irenae*	SLO, Misliče, Susica stream, 06.12.2009	CIxEI0104	JF891172	Dery & Szivak
*Chaetopteryx irenae*	SLO, Misliče, Susica stream, 06.12.2009	CIxEI0105	JF891173	Dery & Szivak
*Chaetopteryx major*	HUN, Mecsek Mts., Vár valley, Pásztor spring 05.11.2010	CMJKB0101	JF891233	Olah, Szivak & Uherkovich
*Chaetopteryx major*	HUN, Mecsek Mts., Vár valley, Pásztor spring 05.11.2010	CMJKB0102	HE858256	Olah, Szivak & Uherkovich
*Chaetopteryx major*	HUN, Mecsek Mts., Vár valley, Pásztor spring 05.11.2010	CMJKB0103	HE858257	Olah, Szivak & Uherkovich
*Chaetopteryx major*	HUN, Mecsek Mts., Vár valley, Pásztor spring 05.11.2010	CMJKB0104	HE858258	Olah, Szivak & Uherkovich
*Chaetopteryx major*	AUT, valley Hottmannsgraben, Unteraspang (Aspang Markt) 19.11.2009	CMJDJ0101	JF891234	Dery & Szivak
*Chaetopteryx marinkovicae*	CRO, Kompanj, 14.11.2009	CMREI0101	JF891174	Kučinić & Vučković
*Chaetopteryx marinkovicae*	CRO, Kompanj, 14.11.2009	CMREI0102	JF891175	Kučinić & Vučković
*Chaetopteryx marinkovicae*	CRO, Kompanj, 14.11.2009	CMREI0103	JF891176	Kučinić & Vučković
*Chaetopteryx marinkovicae*	CRO, Kompanj, 14.11.2009	CMREI0104	JF891177	Kučinić & Vučković
*Chaetopteryx marinkovicae*	CRO, Kompanj, 14.11.2009	CMREI0105	JF891178	Kučinić & Vučković
*Chaetopteryx rugulosa mecsekensis*	HUN, Mecsek Mts., Nagy-Mély valley, Kánya spring, 14.11.2009	CRMKB0101	JF891179	Szivak
*Chaetopteryx rugulosa mecsekensis*	HUN, Mecsek Mts., Vár valley, Pásztor spring, 06.11.2009	CRMKB0201	JF891180	Szivak & Uherkovich
*Chaetopteryx rugulosa mecsekensis*	HUN, Mecsek Mts., Melegmányi valley, Mésztufa spring, 14.11.2009	CRMKB0301	JF891203	Szivak
*Chaetopteryx rugulosa mecsekensis*	HUN, Mecsek Mts., Vár valley, Iharos spring, 06.11.2009	CRMKB0401	JF891204	Szivak
*Chaetopteryx rugulosa noricum*	AUT, Saualpe, Klieningbach stream near Kliening, 21.11.2009	CRNDI0101	JF891187	Dery & Szivak
*Chaetopteryx rugulosa noricum*	AUT, Saualpe, springs of the Klippitzbach stream near Klippitztörl 21.11.2009	CRNDI0201	JF891188	Dery & Szivak
*Chaetopteryx rugulosa noricum*	AUT, Saualpe, springs of the Klippitzbach stream near Klippitztörl 21.11.2009	CRNDI0202	JF891189	Dery & Szivak
*Chaetopteryx rugulosa noricum*	AUT, Saualpe, springs of the Klippitzbach stream near Klippitztörl 21.11.2009	CRNDI0203	JF891219	Dery & Szivak
*Chaetopteryx rugulosa noricum*	AUT, Saualpe, springs of the Klippitzbach stream near Klippitztörl 21.11.2009	CRNDI0204	JF891220	Dery & Szivak
*Chaetopteryx rugulosa noricum*	AUT, Saulape, spring of the Löllingbach stream near Stranach, 21.11.2009	CRNDI0301	JF891190	Dery & Szivak
*Chaetopteryx rugulosa noricum*	AUT, Saulape, spring of the Löllingbach stream near Stranach, 21.11.2009	CRNDI0302	JF891191	Dery & Szivak
*Chaetopteryx rugulosa noricum*	AUT, Saulape, spring of the Löllingbach stream near Stranach, 21.11.2009	CRNDI0303	JF891217	Dery & Szivak
*Chaetopteryx rugulosa noricum*	AUT, Saulape, spring of the Löllingbach stream near Stranach, 21.11.2009	CRNDI0304	JF891218	Dery & Szivak
*Chaetopteryx rugulosa rugulosa*	HUN, Kőszegi Mts., Hörmann spring near Velem, 18.11.2009	CRRDJ0101		Szivak
*Chaetopteryx rugulosa rugulosa*	HUN, Kőszegi Mts., Hörmann spring near Velem, 18.11.2009	CRRDJ0102		Szivak
*Chaetopteryx rugulosa rugulosa*	AUT, Mitterneuwald, Hermann spring, 19.11.2009	CRRDJ0201	JF891184	Dery & Szivak
*Chaetopteryx rugulosa rugulosa*	AUT, Sommeralm, Mixnitzbach stream, 20.11.2009	CRRDJ0301		Dery & Szivak
*Chaetopteryx rugulosa rugulosa*	AUT, Sommeralm, Mixnitzbach stream, 20.11.2009	CRRDJ0302	JF891214	Dery & Szivak
*Chaetopteryx rugulosa rugulosa*	AUT, Hochegg bei Grimmenstein, spring and its outlet, 19.11.2009	CRRDJ0401	JF891205	Dery & Szivak
*Chaetopteryx rugulosa rugulosa*	AUT, Hochegg bei Grimmenstein, spring and its outlet, 19.11.2009	CRRDJ0402	JF891206	Dery & Szivak
*Chaetopteryx rugulosa rugulosa*	AUT, Hochegg bei Grimmenstein, spring and its outlet, 19.11.2009	CRRDJ0403	JF891207	Dery & Szivak
*Chaetopteryx rugulosa rugulosa*	AUT, Ausserneuwald, spring, 19.11.2009	CRRDJ0501	JF891208	Dery & Szivak
*Chaetopteryx rugulosa rugulosa*	AUT, Ausserneuwald, spring, 19.11.2009	CRRDJ0502	JF891209	Dery & Szivak
*Chaetopteryx rugulosa rugulosa*	AUT, Plenzengreith, upper reach of stream Schöcklbach, 20.11.2009	CRRDJ0601	JF891230	Dery & Szivak
*Chaetopteryx rugulosa rugulosa*	AUT, Plenzengreith, upper reach of stream Schöcklbach, 20.11.2009	CRRDJ0602	JF891231	Dery & Szivak
*Chaetopteryx rugulosa rugulosa*	AUT, Plenzengreith, upper reach of stream Schöcklbach, 20.11.2009	CRRDJ0603	JF891232	Dery & Szivak
*Chaetopteryx rugulosa rugulosa*	SLO, Pohorje Mts., Osankarica (Lukanja), 10.11.2008	CRRDG0101	JF891186	Popijač
*Chaetopteryx rugulosa rugulosa*	SLO, Pohorje Mts., Osankarica (Lukanja), 10.11.2008	CRRDG0102	JF891215	Popijač
*Chaetopteryx rugulosa rugulosa*	SLO, Pohorje Mts., Osankarica (Lukanja), 10.11.2008	CRRDG0103	JF891216	Popijač
*Chaetopteryx rugulosa rugulosa*	CRO, Medvednica Mts., Mrzlak spring near Sljeme, 18.11.2006	CRREE0101	JF891185	Popijač
*Chaetopteryx rugulosa rugulosa*	CRO, Medvednica Mts., Mrzlak spring near Sljeme, 18.11.2006	CRREE0102	JF891213	Popijač
*Chaetopteryx rugulosa rugulosa*	CRO, Medvednica Mts., Kraljičin Zdenac spring, Kraljičin Zdenac, 19.11.2009	CRREE0201	JF891210	Kučinić & Vučković
*Chaetopteryx rugulosa rugulosa*	CRO, Medvednica Mts., Bliznec stream, Podsljeme (Pilana), 09.12.2009	CRREE0301	JF891211	Kučinić & Vučković
*Chaetopteryx rugulosa rugulosa*	CRO, Žumberak Mts., Slapnica stream, Ribička kuća, 28.10.2009	CRREF0101	JF891212	Kučinić & Vučković
*Chaetopteryx schmidi*	ROU, spring brook in Cerna valley near Tatu, 13.11.2010	CRSJF0101	HE858259	Ecsedi & Szivak
*Chaetopteryx schmidi*	ROU, spring brook in Cerna valley near Tatu, 13.11.2010	CRSJF0102	HE858260	Ecsedi & Szivak
*Chaetopteryx schmidi*	ROU, spring brook in Cerna valley near Tatu, 13.11.2010	CRSJF0103	HE858261	Ecsedi & Szivak
*Chaetopteryx schmidi*	SRB, Derdap Mts., stream valley N of Golubinje, 13.10.2006	CRSGE0101	JF891182	Danyi, Kontschan & Muranyi
*Chaetopteryx schmidi*	SRB, Derdap Mts., stream valley N of Golubinje, 13.10.2006	CRSGE0102	JF891201	Danyi, Kontschan & Muranyi
*Chaetopteryx schmidi*	SRB, Derdap Mts.,Grgeci spring, Donji Milankovac, 13.10.2006	CRSGE0201	JF891183	Danyi, Kontschan & Muranyi
*Chaetopteryx schmidi*	SRB, Derdap Mts.,Grgeci spring, Donji Milankovac, 13.10.2006	CRSGE0203	JF891202	Danyi, Kontschan & Muranyi
*Chaetopteryx bucari* sp. n.	CRO, Kriz spring near Petrinja, 08.12.2009	CxxEC0101	JF891192	Kučinić, Delić & Bučar
*Chaetopteryx bucari* sp. n.	CRO, Kriz spring near Petrinja, 07.11.2009	CxxEC0102	JF891222	Kučinić, Delić & Bučar
*Chaetopteryx bucari* sp. n.	CRO, Kriz spring near Petrinja, 07.11.2009	CxxEC0103	JF891223	Kučinić, Delić & Bučar
*Chaetopteryx bucari* sp. n.	CRO, Kriz spring near Petrinja, 04.11.2009	CxxEC0104	JF891224	Bučar
*Chaetopteryx bucari* sp. n.	CRO, Kriz spring near Petrinja, 08.12.2009	CxxEC0105	JF891225	Kučinić, Delić, Bučar & Vučković
*Chaetopteryx bucari* sp. n.	CRO, Hrvatski Cuntic, Marića točak spring, 22.11.2009	CxxEC0201	JF891193	Kučinić, Delić & Bučar
*Chaetopteryx bucari* sp. n.	CRO, Hrvatski Cuntic, Marića točak spring, 21.11.2009	CxxEC0202	JF891221	Kučinić, Delić & Bučar
*Chaetopteryx bucari* sp. n.	CRO, Hrvatska Kostajnica, Varoški bunar spring, 06.12.2009	CxxEC0301		Kučinić, Delić & Bučar
*Chaetopteryx bucari* sp. n.	CRO, Šuplji Kamen, Slabinja spring, 29.11.2009	CxxEC0401	JF891194	Kučinić, Delić & Bučar
*Chaetopteryx bucari* sp. n.	CRO, Banovina region, Pecki spring, 15.12.2009	CxxEC0501	JF891195	Kučinić, Delić & Bučar
*Chaetopteryx bucari* sp. n.	CRO, Banovina region, Pecki spring, 21.11.2009	CxxEC0502	JF891228	Kučinić, Delić & Bučar
*Chaetopteryx bucari* sp. n.	CRO, Banovina region, Pecki spring, 21.11.2009	CxxEC0503	JF891229	Kučinić, Delić & Bučar
*Chaetopteryx bucari* sp. n.	CRO, Banovina region, Gora spring, 10.12.2009	CxxEC0601	JF891226	Bučar
*Chaetopteryx bucari* sp. n.	CRO, Mečenčani, Pašino vrelo, 29.11.2009	CxxEC0701	JF891227	Kučinić, Delić & Bučar
*Chaetopterygopsis maclachlani*	AUT, Lower Austria, Rohrwiesteich, 20.10.2004	08HMCAD-331*	HMTRI331-09*	Malicky
*Chaetopteryx fusca*	AUT, Lower Austria, Rohrwiesteich, 20.10.2004	08HMCAD-333*	HMTRI333-09*	Malicky
*Chaetopteryx gessneri*	ITA, Umbria, Perugia, Fium Nera above Visso,11.12.2005	07HMCAD-0177*	HMCAD177-08*	Malicky
*Chaetopteryx moretti*	ITA, Belluno, Val Canzoi, Veneto, 31.10.2003	HM09Cm7*	HMTRI421-09*	Malicky
*Chaetopteryx villosa*	AUT, Lower Austria, Sarleinsbach, 27.06.2005	07HMCAD-0134*	HMCAD134-08*	Malicky
*Drusus alpinus*	IT, Valprato Soana, Ronchietto, 10.07.2004	HM09Dalp8*	HMTRI456-09*	Delmaistro
*Drusus discolor*	SK, Lower Tatra, Stream above Partizanska L'upča, 09.06.2008	ESCAD909-17*	KKCAD497-09*	Bonada
*Drusus rectus*	ES, Camprodon/Setcases Alta Val de Ter, 27.07.2004	HM09Drec8*	HMTRI423-09*	Aistleitner
*Metanoea rhaetica*	AUT, Carinthia, Valentinbach, Plockenstrasse, 08.07.2007	08HMCAD-020*	HMTRI020-08*	Malicky
*Limnephilus centralis*	NORWAY	NHRS:FI9	FN601020	Malm & Johanson 2011

The mitochondrial COI barcodes were generated at the Canadian Centre for DNA Barcoding, University of Guelph, Canada. Standard barcoding protocols for DNA extraction (Ivanova et al. 2006), PCR amplification and COI sequencing (Hajibabaei et al. 2005, de Waard et al. 2008) were used. Full-length COI-5P DNA barcodes were amplified using C_LepFolF/C_LepFolR (Folmer et al. 1994, Hajibabaei et al. 2006) and LCO1490/HCO2198 (Folmer et al. 1994) primer sets. COI barcodes and detailed specimen information can be found in the Barcode of Life Data Systems (BOLD; http://www.boldsystems.org/) (Ratnasingham and Hebert 2007) within the project “*Chaetopteryx* of Europe.” Unpublished COI barcodes of additional *Chaetopteryx* outgroups were provided by Karl Kjer, Rutgers University, USA (Table 2). The sequence of *Limnephilus centralis* Curtis, 1834 was taken from Malm and Johanson (2011) (Table 2).

**Phylogenetic analysis.** Sequences were edited manually and aligned using the program Geneious 5.4 (Drummond et al. 2011). The final alignment was 617 base pairs (bp) long. Bayesian phylogenetic analyses were performed using the Markov chain Monte Carlo method (B/MCMC) using MrBayes 3.2 (Buckley et al. 2002, Ronquist and Huelsenbeck 2003). We selected the best-fitting models of DNA substitution using Akaike information criterion (AIC) implemented in jModelTest 0.1.1 (Guindon and Gascuel 2003, Posada 2008). jModelTest indicated a general time reversible model (Rodríguez et al. 1990) with a significant proportion of invariant sites (I=0.607) and with gamma-distributed rate heterogeneity (α=1.049) (GTR+I+G). We conducted Bayesian tree construction with 6 chains, 2 independent runs and 8 million generations. Trees were sampled every 1000th generation. The first 9000 generations were discarded as burn-in. We plotted the log-likelihood scores of sample points against generation time using Tracer 1.5 (Rambaut and Drummond 2009) to ensure that stationary was achieved after the first 9000 generations by checking whether the log-likelihood values of the sample points reached a stable equilibrium plateau. We used the remaining trees with average branch lengths to create a 50% majority-rule consensus tree with the sumt option of MrBayes. Posterior probabilities (pp) were obtained for each clade, whereby pp≥0.95 indicated significant support for clades. Finally, we also calculated the uncorrected pairwise distances between individuals based on mtCOI sequences using MEGA 5.1 (Tamura et al. 2011).

**Microphotography and measuring.** Microphotographic images of genitalia and forewing measurements were taken using a Leica Wild MZ8 stereomicroscope and Olympus SP-500 UZ digital camera. The photographs were processed with the Olympus Quick Photo Camera 2.2. software package. Geographic coordinates and altitudes of sampling localities were recorded with a Garmin ‘Oregon 450' GPS device.

## Results

**Figure 2.**
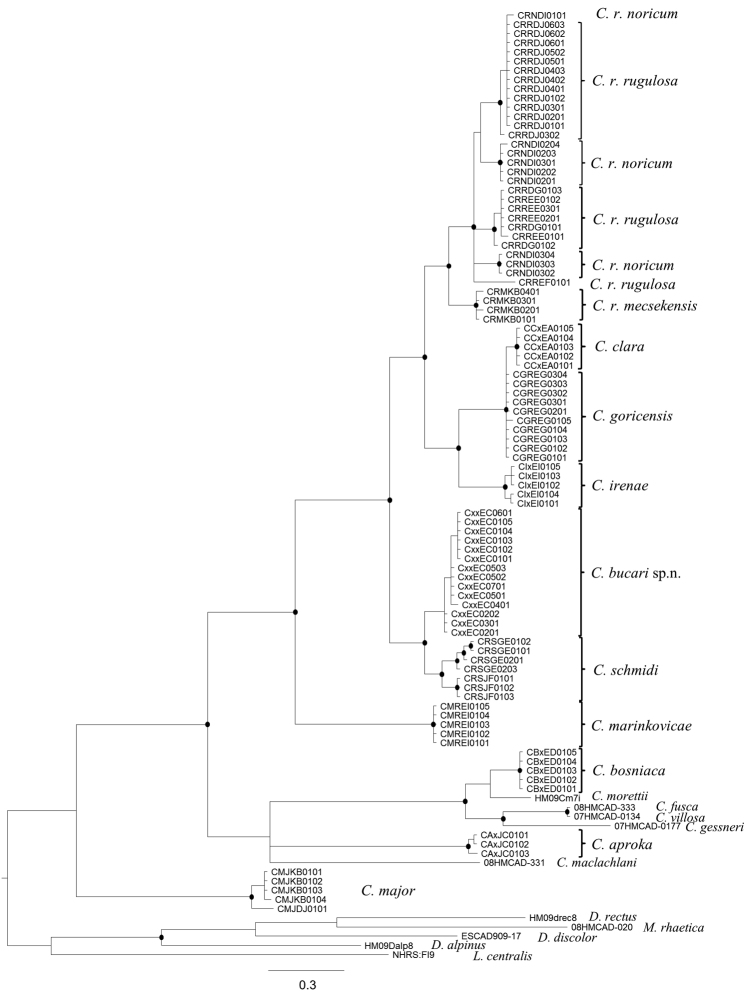
Bayesian tree for members of the *Chaetopteryx rugulosa* species group based on mitochondrial COI sequence. Black circles on nodes mark Bayesian posterior probabilities pp>0.95.

**Phylogenetic analyses.** In the Bayesian phylogenetic tree based on mtCOI sequences the *Chaetopteryx rugulosa* group species clustered into 4 strongly supported clades (Figure 2). *Chaetopteryx marinkovicae* was basal within the species group. The remaining species fell into 3 clades: a basal clade with *Chaetopteryx rugulosa schmidi*, *Chaetopteryx bucari* sp. n., and 2 derived sister clades comprising *Chaetopteryx clara*, *Chaetopteryx goricensis*, *Chaetopteryx irenae*, and *Chaetopteryx rugulosa rugulosa*, *Chaetopteryx rugulosa noricum*, *Chaetopteryx rugulosa mecsekensis*. *Chaetopteryx bucari* sp. n. is sister to the highly supported *Chaetopteryx rugulosa schmidi*. The mean value of the uncorrected pairwise distance (p distance) was 2.02% between them (Table 3). The p distance did not reach 1% within the 2 clades (*Chaetopteryx bucari* sp. n.: 0.17%; *Chaetopteryx rugulosa schmidi*: 0.75%). The relationship of the nominal species of the group *Chaetopteryx rugulosa rugulosa* and *Chaetopteryx rugulosa noricum* was not resolved, as the 4 subclades formed a polytomy. In the phylogenetic tree *Chaetopteryx rugulosa schmidi* was clearly separated from the clade containing the subspecies of *Chaetopteryx rugulosa* (Figure 2). The mean values of p distance between the 3 subspecies of *Chaetopteryx rugulosa* ranged between 1.61–3.02 %, while the mean values between the *Chaetopteryx rugulosa schmidi* and the other subspecies of *Chaetopteryx rugulosa* were distinctly higher (4.66 – 5.85%) (Table 3).

**Table 3.** Estimates of evolutionary divergence over sequence pairs within and between phylogenetically defined species and subspecies based on mtCOI sequence data. Distance matrix is shown with the mean ± SD values of the intraspecific and interspecific pairwise genetic distances for the all Chaetopterygini species included in the analysis. Abbrev.: CRR – *Chaetopteryx rugulosa rugulosa*, CRN – *Chaetopteryx rugulosa noricum*, CRM – *Chaetopteryx rugulosa mecsekensis*, CCX – *Chaetopteryx clara*, CGR – *Chaetopteryx goricensis*, CIX – *Chaetopteryx irenae*, CBU – *Chaetopteryx bucari* sp.n., CRS – *Chaetopteryx schmidi*, CMR – *Chaetopteryx marinkovicae*, CBA – *Chaetopteryx bosniaca*, CMO – *Chaetopteryx morettii*, CFU – *Chaetopteryx fusca*, CVI – *C. villosa*, CGE – *Chaetopteryx gessneri*, CAX – *Chaetopteryx aproka*, CMA – *Chaetopterygopsis maclachlani*, CMJ – *Chaetopteryx major*.

	**CRR**	**CRN**	**CRM**	**CCX**	**CGR**	**CIX**	**CBU**	**CRS**	**CMR**	**CBO**	**CMO**	**CFU**	**CVI**	**CGE**	**CAX**	**CMA**	**CMJ**
**CRR**	1.05±0.97	1.61±0.49	3.02±0.17	4.87±0.24	4.55±0.24	4.79±0.27	4.63±0.30	5.44±0.39	9.24±0.46	12.64±0.15	12.85±0.33	12.69±0.20	12.69±0.20	14.03±0.20	12.00±0.39	12.32±0.20	14.17±0.16
**CRN**		1.20±0.89	2.83±0.31	5.06±0.23	4.74±0.24	5.26±0.20	5.17±0.18	5.85±0.11	9.45±0.27	12.85±0.08	12.66±0.12	12.83±0.12	12.83±0.12	14.06±0.12	12.18±0.14	11.98±0.24	14.14±0.39
**CRM**			0.17±0.11	4.79±0.09	4.47±0.10	4.69±0.12	4.38±0.11	4.66±0.13	8.87±0.07	11.89±0.07	11.97±0.08	11.76±0.14	11.76±0.14	12.44±0.14	11.60±0.12	11.47±0.08	13.78±0.00
**CCx**				0.00±0.00	0.37±0.07	3.80±0.08	5.86±0.14	5.69±0.06	9.92±0.00	12.61±0.00	12.61±0.00	12.77±0.00	12.77±0.00	13.61±0.00	12.77±0.00	12.61±0.00	14.49±0.07
**CGR**					0.03±0.07	3.48±0.10	5.54±0.15	5.37±0.08	9.93±0.05	12.62±0.05	12.62±0.05	12.79±0.05	12.79±0.05	13.63±0.05	12.78±0.05	12.62±0.05	14.17±0.08
**CIX**						0.10±0.09	4.41±0.16	5.11±0.18	10.15±0.08	12.67±0.08	13.21±0.09	12.54±0.09	12.54±0.09	13.04±0.09	13.38±0.09	13.55±0.09	15.39±0.11
**CBa**							0.17±0.13	2.02±0.20	8.79±0.13	12.48±0.13	12.65±0.14	12.48±0.14	12.48±0.14	12.65±0.14	11.21±0.08	12.89±0.08	14.00±0.16
**CRS**								0.75±0.57	8.72±0.06	12.24±0.06	13.04±0.13	12.05±0.21	12.05±0.21	12.67±0.23	11.56±0.22	13.93±0.06	14.44±0.15
**CMR**									0.00±0.00	11.60±0.00	11.60±0.00	11.93±0.00	11.93±0.00	13.11±0.00	11.76±0.00	12.94±0.00	12.77±0.18
**CBO**										0.00±0.00	2.69±0.00	4.87±0.00	4.87±0.00	6.05±0.00	12.61±0.00	12.61±0.00	15.26±0.17
**CMO**											0.00±0.00	5.55±0.00	5.55±0.00	6.55±0.00	12.94±0.00	11.76±0.00	14.82±0.08
**CFU**												0.00±0.00	0.00±0.00	5.88±0.00	13.05±0.19	12.94±0.00	15.83±0.08
**CVI**													0.00±0.00	5.88±0.00	13.05±0.19	12.94±0.00	15.83±0.08
**CGE**														0.00±0.00	13.45±0.00	13.44±0.00	15.87±0.09
**CAX**															0.35±0.00	11.71±0.19	13.44±0.11
**CMA**																0.00±0.00	13.04±0.15
**CMJ**																	0.47±0.51

### Chaetopteryx bucari

Kučinić, Szivák & Delić sp. n.

href="http://zoobank.org/E775EC69-0E8A-4AF0-A027-F290BB31E76E

http://species-id.net/wiki/Chaetopteryx_bucari

Figures 3 –16

#### Type material.

**Holotype male:** CROATIA, Pecki spring, 45°23'50"N, 16°14'40"E, 161 m a.s.l., 15 December 2009, leg. Bučar, Delić, Kučinić, dry specimen, DNA Barcode ID: HGCAD046-10, deposited in the Croatian Natural History Museum in Zagreb.

**Paratype**: CROATIA, ♂ and ♀ (n=49): 1 female, Pecki spring, 21 November 2009, leg. Bučar, Delić, Kučinić, dry specimen, DNA Barcode ID: HGCAD087-10; 14 males, Pecki spring, 31 October 2011; 9 females, Pecki spring, 31 October 2010; 20 females, Pecki spring, 30 November 2011; 2 males and 2 females, Hrvatski Čuntić stream, 45°21'28"N, 16°17'04"E, 159 m a.s.l., 22 October 2010; 1 male, Marića točak, 45°21'29"N, 16°17'03"E, 163 m a.s.l., 23 November 2012, leg. Bučar, Delić, Kučinić (all specimens in alcohol).

#### Diagnosis.

Male of *Chaetopteryx bucari* is most similar to *Chaetopteryx rugulosa mecsekensis* and *Chaetopteryx rugulosa schmidi* but differs in the following features: 1. In lateral view the inferior appendages in *Chaetopteryx bucari* are always with a pointed apex on the dorsal side, not rounded as in *Chaetopteryx rugulosa mecsekensis*; 2. Bristles in *Chaetopteryx bucari* are set more distally from the membranous part of the aedeagus than in *Chaetopteryx rugulosa mecsekensis* and *Chaetopteryx rugulosa schmidi* and never reach (touch) the lateral membranous finger, as in *Chaetopteryx rugulosa mecsekensis*. Female of *Chaetopteryx bucari* is clearly different from other species in the *Chaetopteryx rugulosa* group (e.g., form of the visible finger on lateral side, form of the anal tube, form of the supragenital plate of segment X in lateral and ventral views, form of the median lobe of the vulvar scale in ventral view). We did not find strong morphological variability among the females of the new species (except the median lobe of the vulvar scale). Females of *Chaetopteryx bucari* have in lateral, ventral and dorsal views very visible finger-shaped proturbances (ventral lobes of tergite IX) on the anal tube which is lacking in *Chaetopteryx rugulosa mecsekensis* and *Chaetopteryx rugulosa schmidi*. In lateral view the excision of the anal tube in *Chaetopteryx rugulosa rugulosa* is more pronounced than in *Chaetopteryx bucari*. The median lobe of the vulvar scale in *Chaetopteryx rugulosa mecsekensis*, *Chaetopteryx rugulosa rugulosa* and *Chaetopteryx rugulosa schmidi* is longer and more visible than in *Chaetopteryx bucari*.

#### Description.

**Figure 3.**
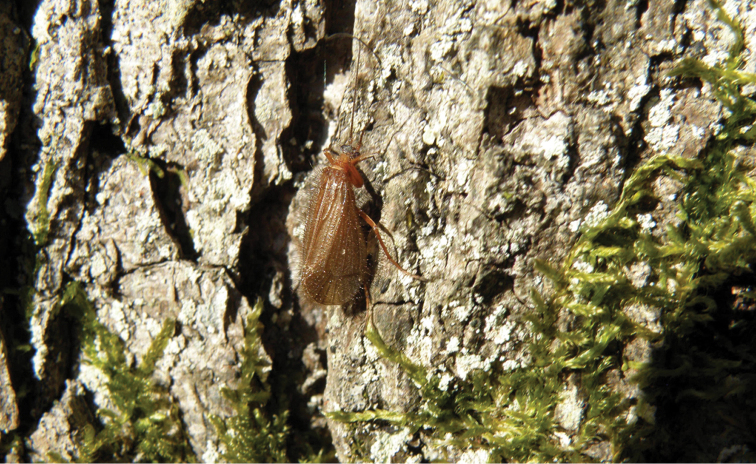
*Chaetopteryx bucari* sp. n., adults at type locality, Pecki spring, Croatia.

Wings and legs yellow to yellowish-brown; veins darker in both sexes (Figure 3). Antennae long, grey to fuscous. Scapus yellow to yellowish-brown, thorax and abdomen yellow. Spur formula male 0,3,3, female 1,3,3. Ocelli present. Forewing with round apex; length 7.7–9.9 mm in males, 7.2–10.1 mm in females.

**Figure 4.**
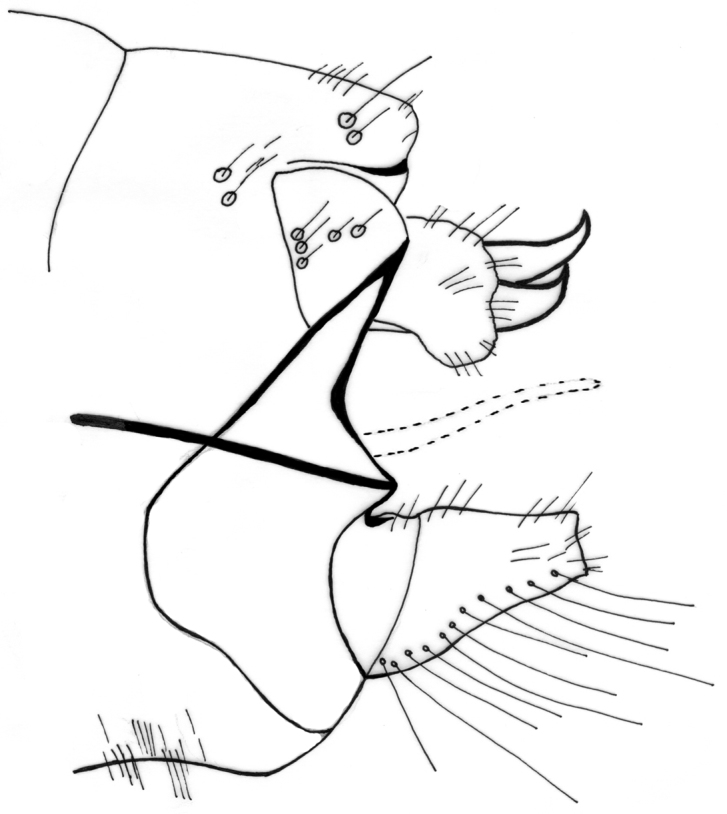
*Chaetopteryx bucari* sp. n., male genitalia, lateral view.

**Figure 5.**
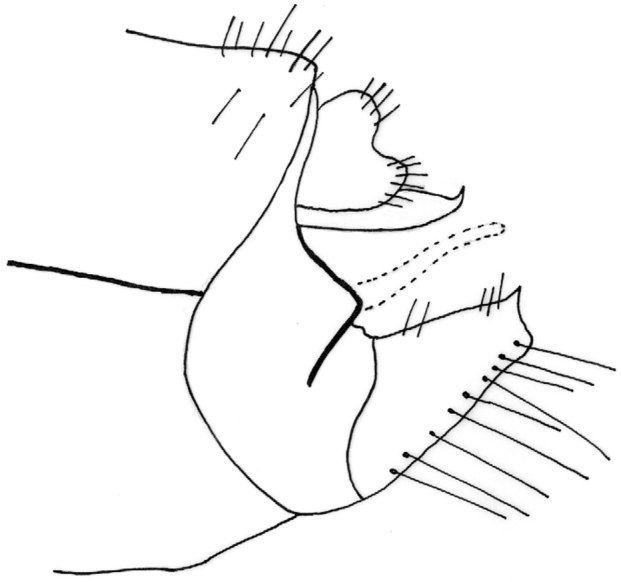
*Chaetopteryx bucari* sp. n., male genitalia, lateral view.

**Figure 6.**
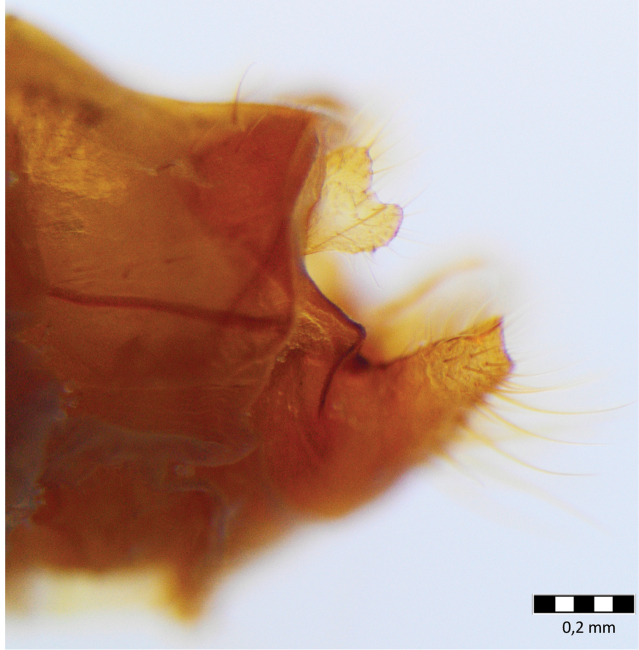
*Chaetopteryx bucari* sp. n., male genitalia, lateral view.

**Figure 7.**
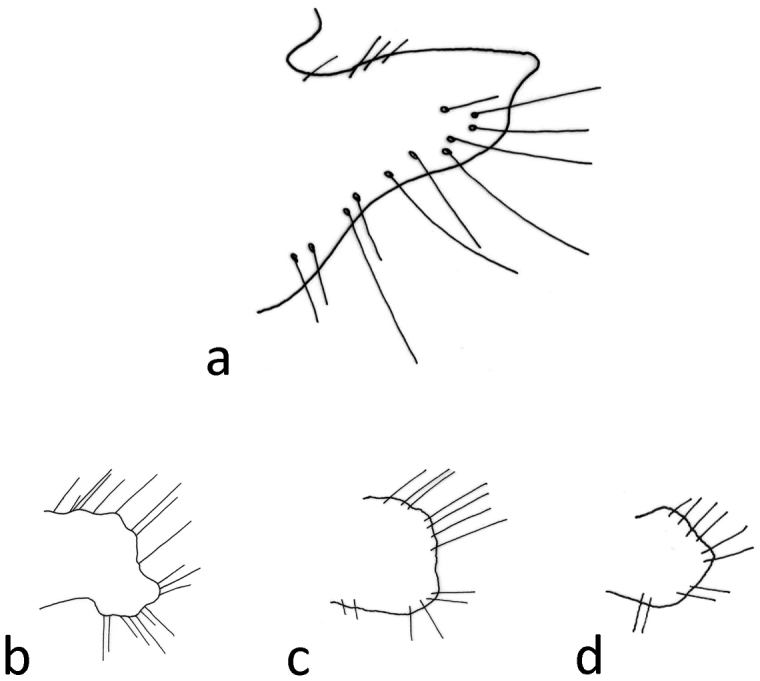
*Chaetopteryx bucari* sp. n., male genitalia, lateral view **a** inferior appendages **b–d** superior appendages.

**Figure 8.**
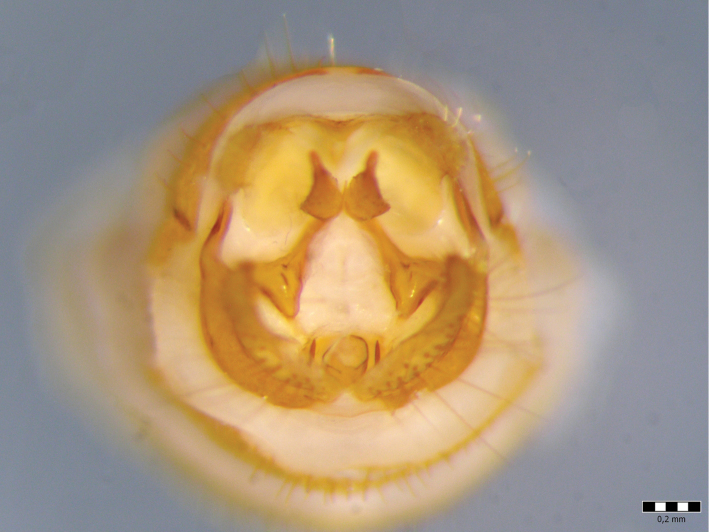
*Chaetopteryx bucari* sp. n., male genitalia, caudal view.

**Figure 9.**
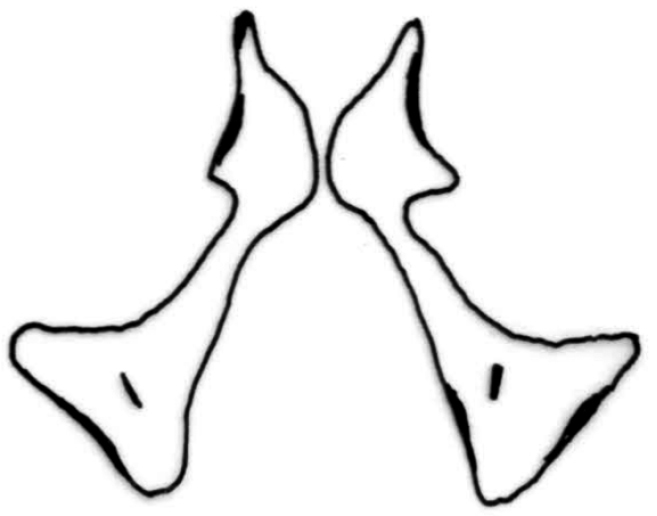
*Chaetopteryx bucari* sp. n., male genitalia, intermediate appendages (paraproctal complex), caudal view.

**Figure 10.**
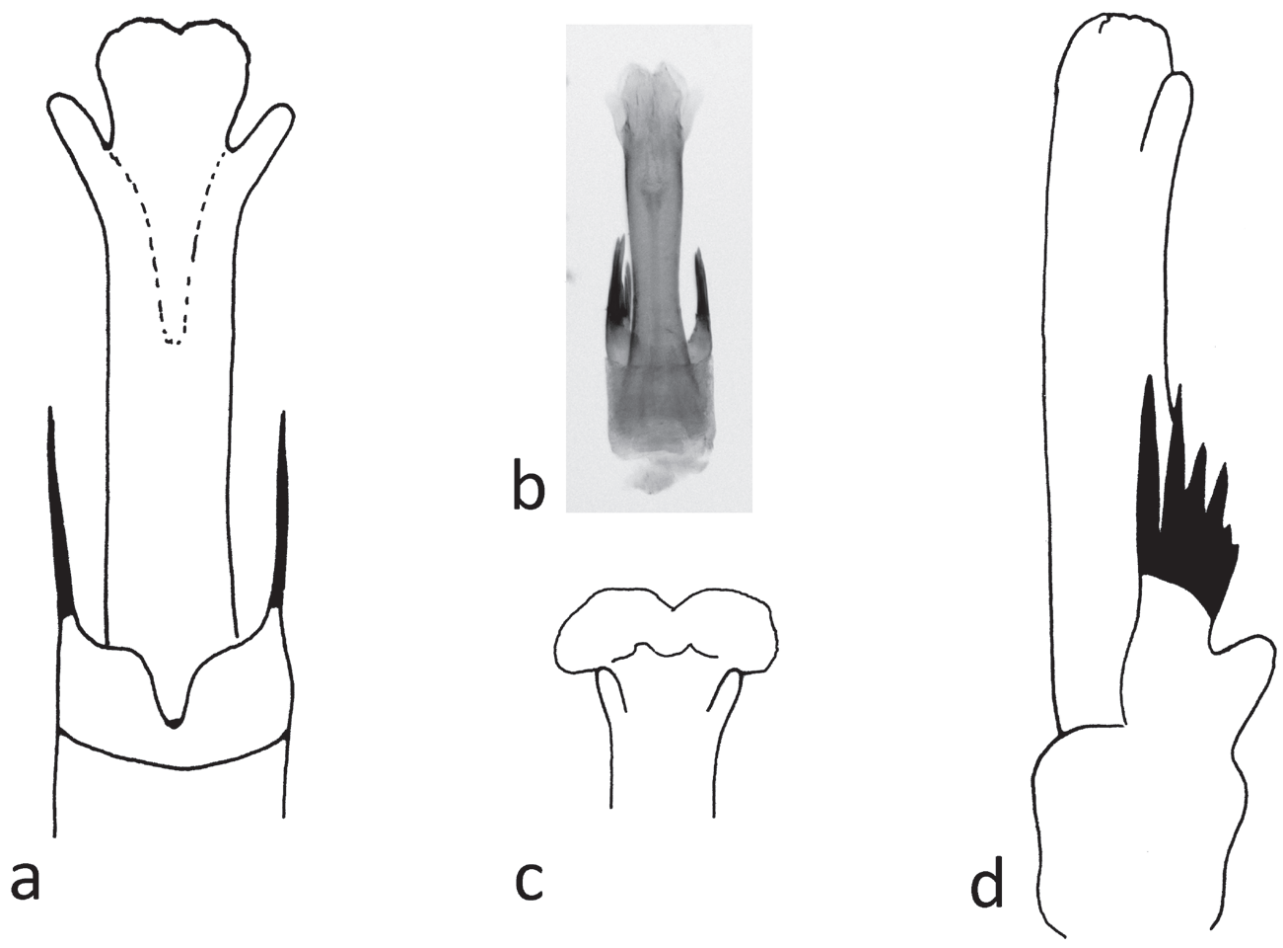
*Chaetopteryx bucari* sp. n., male genitalia, phallic organ (phallus): **a** dorsal view **b** ventral view **c** posterior membranous part of aedeagus **d** lateral view.

**Figure 11.**
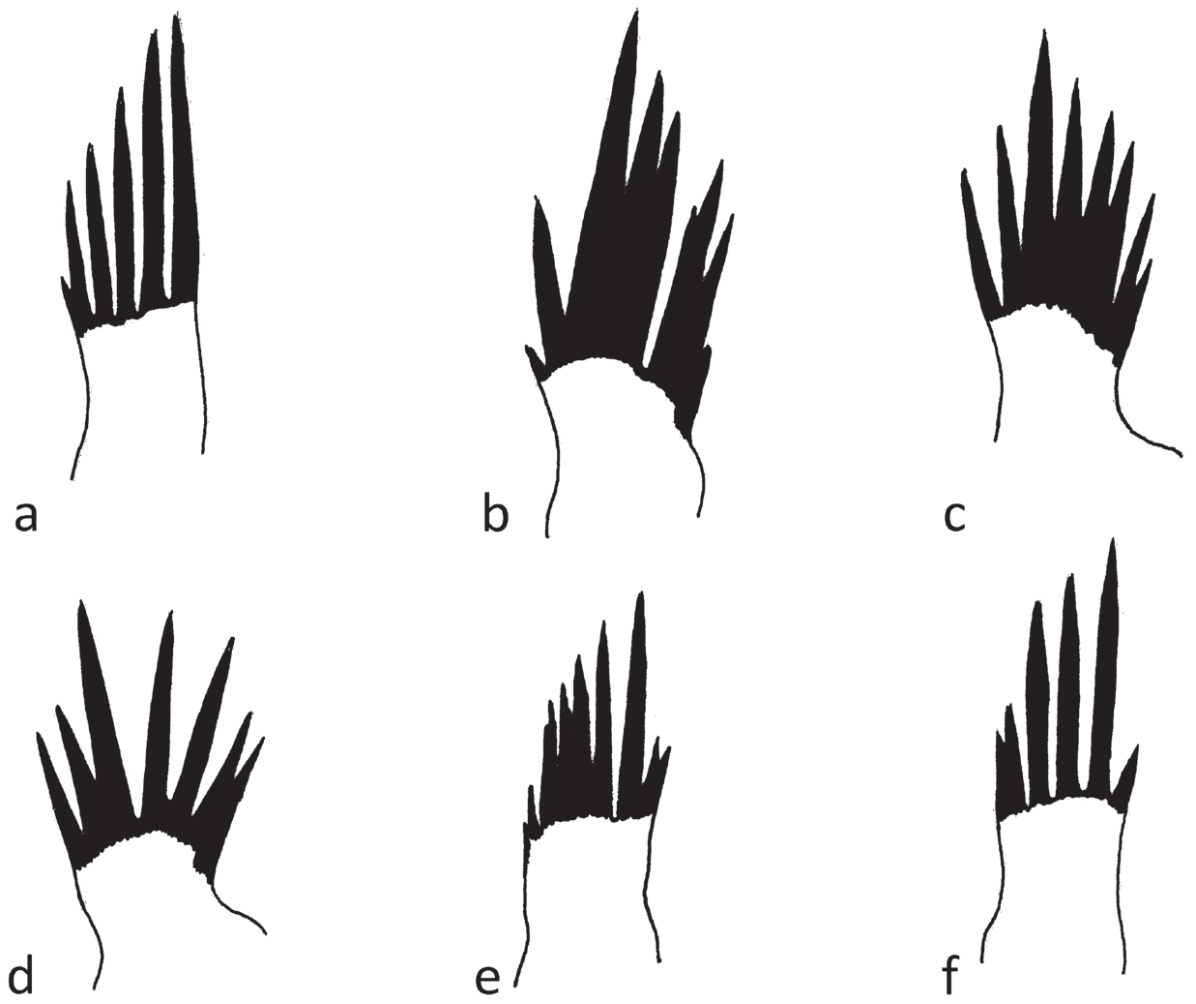
*Chaetopteryx bucari* sp. n., male genitalia **a-f** parameres with sclerotized bristles.

Male genitalia (Figures 4–11). In dorsal view, spinulose zone of tergite VIII well developed with yellow setae. Segment IX ventrally broad, dorsally narrow in lateral view (Figures 4–5). Superior appendages with small yellow setae, shape of superior appendages variable (Figures 4–7b–d), usually in one of two forms (Figures 4–6). In lateral view, 1st form with posterior edge slightly rounded apically, concave at middle (Figure 5); in 2nd form, dorsal side more protuberant with round or irregular apex (Figures 4, 7b). In some specimens triangular or rectangular intermediate forms are found (Figure 7c-d). Inferior appendages in lateral view rectangular, anterior part broad, posterior part narrow (Figures 4–7a). Apical flap of inferior appendage developed, in lateral view with pointed apex (tip) and ventral side slightly rounded; or with apex forked, long setae present on ventral side (Figures 4–7a). Intermediate appendages (paraproctal complex) elongated in lateral view with long, connecting middle section, apical hook narrowing with upward–curving apex (Figures 4–5), basal triangular part of paraproct relatively large in caudal view (Figures 8–9). Phallic organ (phallus) a single tube consisting of phallic apodeme, phallobase, aedeagus and parameres. Aedeagus relatively long, sclerotized, in posterior part with membranous lobes, lateral lobes membranous finger-like proturbances (endophallus) (Figures 10a–d). Two relatively short parameres set very distant from posterior membranous part of aedeagus (Figures 10a–b, 10d); parameres with sclerotized, straight, stout, brown bristles (Figures 10a–b, 10d, 11a–f). Bristles vary in width and length (Figure 11a–f); lateral bristles shorter; bristles arranged in 1 fan-like row (Figure 11a–f); in specimens with more bristles, some form 2nd row; bristles vary from 5-10.

**Figure 12.**
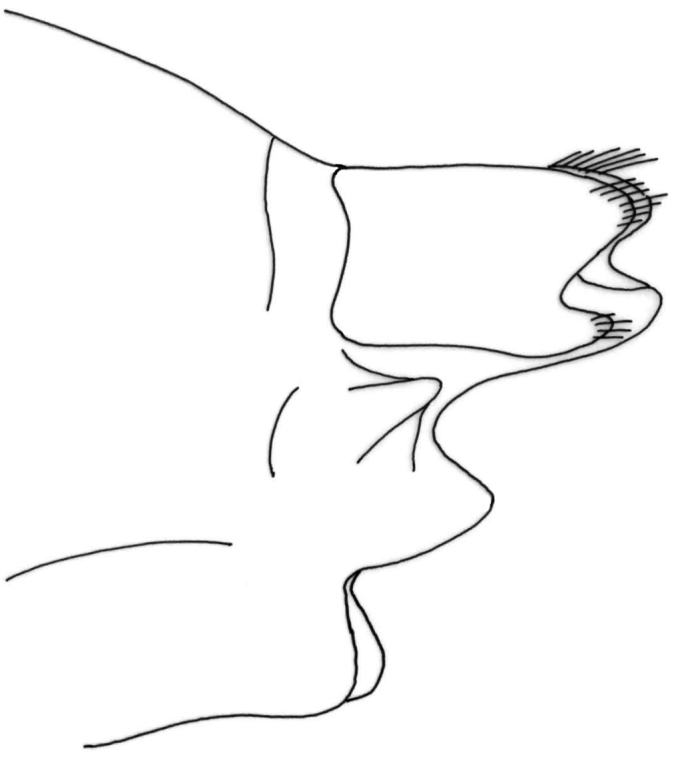
*Chaetopteryx bucari* sp. n., female genitalia, lateral view.

**Figure 13.**
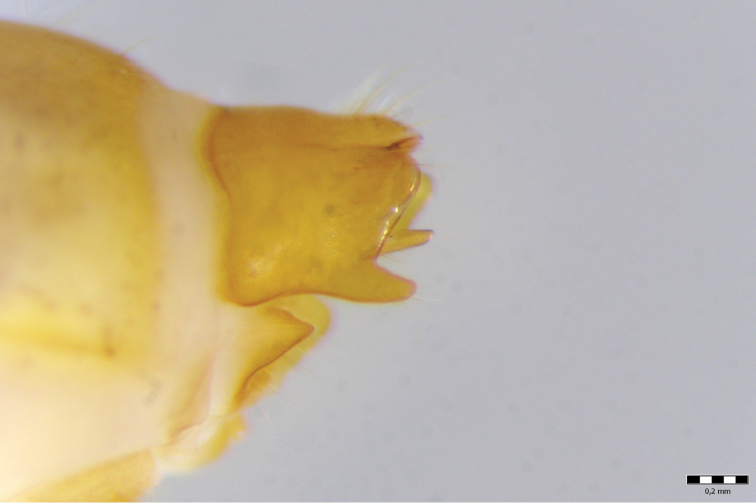
*Chaetopteryx bucari* sp. n., female genitalia, dorso-lateral view.

**Figure 14.**
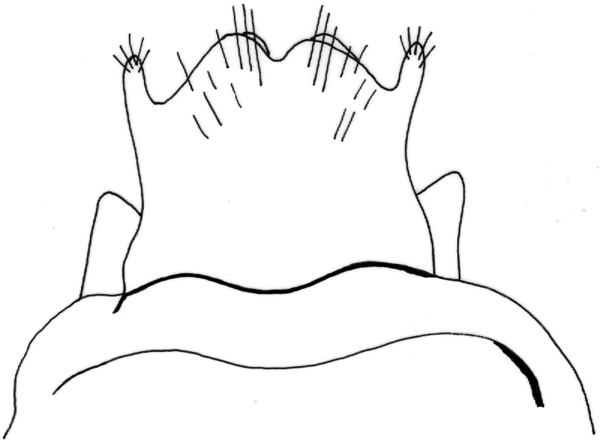
*Chaetopteryx bucari* sp. n., female genitalia, dorsal view.

**Figure 15.**
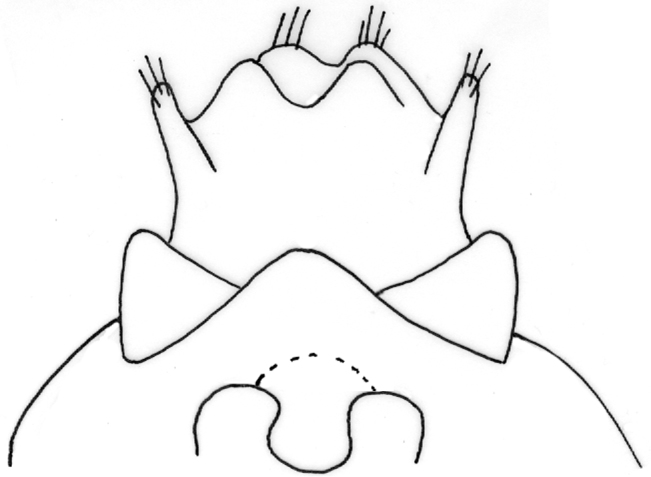
*Chaetopteryx bucari* sp. n., female genitalia, ventral view.

**Figure 16.**
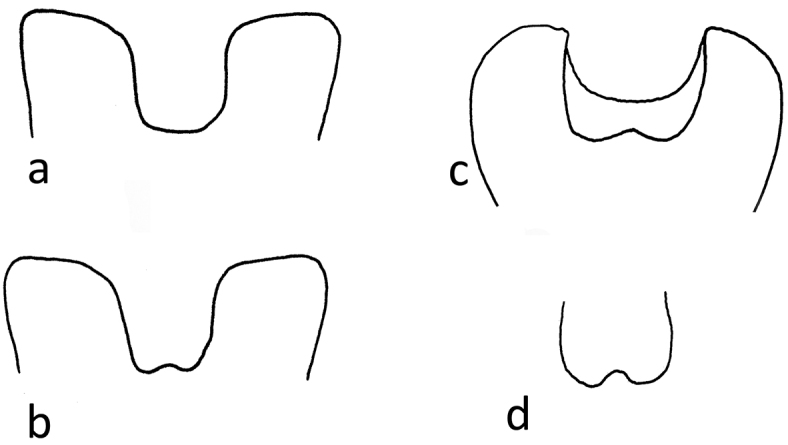
*Chaetopteryx bucari* sp. n., female genitalia**a–d** vulvar scale and median lobe of vulvar scale, ventral view.

Female genitalia (Figures 12–16). Anal tube (fusion of tergites IX and X) in lateral view broad, relatively elongated with one excision and very distinct finger-shaped proturbance (lobes of tergite IX) on ventral side (Figures 12–13). Apex of proturbance rounded or slightly pointed with small yellow setae (Figures 12–15). In 2/3rds of specimens examined ventral and dorsal lips of anal tube equal in length, in 1/3rd ventral lip longer. In dorsal view anal tube thickened with digitate proturbance on lateral side and small excision (recess) in middle (Figure 14). In ventral view anal tube broad with larger excision (recess) in middle than in dorsal side (Figure 15). Supragenital plate of segment X well-developed, triangular in shape in lateral and ventral views (Figures 12, 15). Lateral segment of vulvar scale relatively short in ventral view, with flat or slightly rounded apex (Figure 16a–c). Median lobe of vulvar scale (lower vulvar lip) with very small rounded or pointed apex (Figure 16b–d). In ca. 1/3rd of specimens' median lobe of vulvar scale not visible (Figure 16a).

#### Etymology.

The species is dedicated to Professor Matija Bučar from the Faculty of Education, Department in Petrinja, University of Zagreb.

#### Ecological notes and distribution.

During our recent faunal surveys in Croatia and the Western Dinaric Balkan *Chaetopteryx bucari* was found only at 8 localities in the Banovina region (Table 1). The most distant sampling sites are 40 km apart (Slabinja and Gore). We collected *Chaetopteryx bucari* from 2 springs, 5 wellsprings and 1 location in the stream (Table 1). In total, we collected more than 580 specimens of *Chaetopteryx bucari* (85% were collected in pyramid-type emergence traps). The most abundant populations were found at Pecki spring and a headwater stream in Hrvatski Ćuntić. Over 150 specimens of *Chaetopteryx bucari* were observed on the night of October 14, 2010 on the walls of a small building next to the stream in Hrvatski Čuntić. In Pecki spring more than 50 specimens were observed on the night of October 31, 2010. *Chaetopteryx bucari* was recorded at low altitudes between 104–185 m a.s.l. (Table 1).

*Chaetopteryx bucari* was collected in pyramid-type emergence traps from the end of September-December. The highest number of specimens was collected in October and November in both years. The sex ratio in both years was biased toward males, 1:1.37 (♀♀: ♂♂) in 2010, and 1:1.40 (♀♀:♂♂) in 2011. Besides *Chaetopteryx bucari*, *Chaetopteryx gonospina* Marinković-Gospodnetić, 1966 and 2 additional caddisfly species (*Limnephilus rhombicus* (Linnaeus, 1758), *Potamophylax pallidus* Klapálek, 1898) were recorded in the emergence traps.

In addition to *Chaetopteryx bucari* 2 other species of the *Chaetopteryx rugulosa* group were collected in Croatia during our recent surveys. *Chaetopteryx marinkovicae* was collected from its type locality on the stream and spring in Kompanj village (Istria region); *Chaetopteryx rugulosa rugulosa* was caught on Mt. Žumberak and Mt. Medvednica (northeast and central Croatia). Other species of *Chaetopteryx* found during this investigation were *Chaetopteryx bosniaca* Marinković-Gospodnetić, 1959 (Lika region), *Chaetopteryx gonospina* Marinković-Gospodnetić, 1966 (Banovina region), *Chaetopteryx fusca* (central Croatia, Dalmatia and Lika regions), and *Chaetopteryx major* (central Croatia).

## Discussion

**Systematic and taxonomic implications.** Based on molecular evidence, we could confirm the hypothesis that *Chaetopteryx bucari* is a distinct species. Although *Chaetopteryx bucari* does not have a pp>0.95, it represents the sister taxon (pp>0.95) to the highly supported *Chaetopteryx rugulosa schmidi*. Furthermore, the mean genetic distance (2.02%) between *Chaetopteryx bucari* and *Chaetopteryx rugulosa schmidi* barely reached the 2-3% divergence observed as an interspecific genetic divergence in mtCOI sequences among some well-defined caddisfly species (Bálint et al. 2009, Pauls et al. 2009, Kučinić et al. 2011). However, among other well-defined caddisfly species this value can reach much higher levels (e.g., Zhou et al. 2007, Pauls et al. 2010), but also much lower values (e.g., Waringer et al. 2007). Thus reliance on distance methods alone for defining species boundaries is not advisable and species boundaries should be supported by additional lines of evidence such as additional, independent genes, morphology, or other independent characteristics (Zhou et al. 2007), particularly in taxa where hybridization is possible as is the case in *Chaetopteryx* (Malicky et al. 1986, Malicky and Pauls 2012). In the present study the genetic distinctiveness of *Chaetopteryx bucari* in combination with differences in morphological characters compared to its congeners, provide strong evidence to justify describing itas a new species.

In both sexes, especially in the adult female, *Chaetopteryx bucari* is relatively easily distinguishable from other taxa of the *Chaetopteryx rugulosa* group. The genetic data also show that specimens from 7 populations across the known range of the species from a clearly distinct clade from all other analysed *Chaetopteryx*. It is interesting that the female of *Chaetopteryx bucari* is particularly informative in diagnosing the species. In caddisflies this is quite unusual as males are generally more easily distinguished and females are often very difficult to differentiate from one another.

Based on the phylogenetic position of *Chaetopteryx rugulosa schmidi* in relation to *Chaetopteryx rugulosa rugulosa* and the other *Chaetopteryx rugulosa* subspecies, *Chaetopteryx rugulosa schmidi* is well-defined and quite divergent from other members of the *Chaetopteryx rugulosa* clade based on molecular data. Thus, the subspecies *Chaetopteryx rugulosa schmidi* is here re-established as a distinct species, *Chaetopteryx schmidi*, as it was described originally by Botosaneanu (1957) (Table 2) and not recognized as a subspecies of *Chaetopteryx rugulosa* as proposed by Malicky (2004, 2005).

**Ecology.** The emergence pattern of *Chaetopteryx bucari* corresponds with the general autumnal emergence patterns of the genus, usually from September-December, though emergence can be prolonged through January for some *Chaetopteryx* species (Kučinić 2002), including *Chaetopteryx bucari* (some specimens were collected by handpicking during January 2011). The emergence data from 2 years revealed that the sex ratio of *Chaetopteryx bucari* at the spring of Pecki stream is not exactly 1:1, but biased towards a surplus of males. In other studies applying the same methodology only a few species had 1:1 sex ratios (Kučinić 2002). In some species the sex ratio was 1:6 in favour of females (Previšić et al. 2007) and in other species males were dominant (Kučinić 2002, Semnički et al. 2011). These results are influenced by biological features of the species (e.g., emergence, oviposition behaviour of females), but may also be affected by trapping method (e.g., types of emergence pyramid-traps) (Malicky 2002).

Research on the diversity of large karst springs on the Balkan Peninsula has revealed high levels of caddisfly diversity. In some cases more than 20 species were collected from a single spring (Marinković-Gospodnetić 1979, Kučinić et al. 2008). This high alpha diversity of large karst springs does not, however, diminish the faunal significance of smaller springs. These are usually characterized by a small number of species, but often these species are highly specialized or local endemic species, such as *Chaetopteryx bucari* at the Pecki spring.

**Figure 17.**
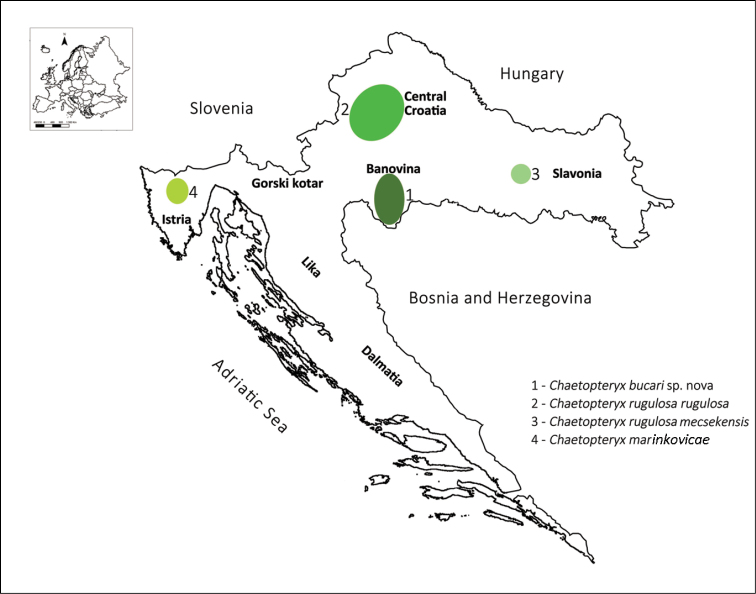
Distribution of *Chaetopteryx rugulosa* group in Croatia.

**Distribution of *Chaetopteryx rugulosa* group in Croatia.** At present, the genus *Chaetopteryx* is represented by 9 taxa in Croatia (Marinković-Gospodnetić 1979, Malicky and Krušnik 1988, Malicky 1996, 2004, Kučinić 2002, Kučinić et al. 2010, Previšić and Popijač 2010, Oláh 2010, 2011a). Including the new species *Chaetopteryx bucari*, 4 species from the *Chaetopteryx rugulosa* group (Malicky and Krušnik 1988, Malicky 1996, 2004, Oláh 2010) are now known from Croatia (Figure 17). Rare species from the genus *Chaetopteryx* are *Chaetopteryx uherkovichi* Oláh, 2011 distributed in eastern Croatia (Slavonia region) so far recorded only at the type locality (Oláh 2011a), *Chaetopteryx rugulosa mecsekensis* known from only 1 locality in Croatia (Malicky 1996, 2004, Oláh 2010), but also distributed in Hungary (Malicky et al. 1986, Malicky 2004) and Serbia (Oláh 2010), and *Chaetopteryx marinkovicae* established in 3 localities in Istria (Malicky and Krušnik 1988). Our research did not confirm the presence of the latter species in 2 of these localities (Malicky and Krušnik 1988), but found specimens at the type locality in Kompanj village. *Chaetopteryx marinkovicae* is also known from Slovenia (Urbanič 2004).

Until now, the new species *Chaetopteryx bucari* was found only in the Banovina region, which is situated between rivers Sava and Kupa to the north and the state border with Bosnia and Herzegovina to the south and east (Figure 17). The Banovina region is characterised by rolling hills up to 600 m a.s.l. There are many small springs and streams in the region, and 3 large rivers, Una, Kupa and Sava, that form the border of the region. It is possible that *Chaetopteryx bucari* is also distributed in some other parts of continental Croatia or in Bosnia and Herzegovina, because we found this species in the valley of the Una River (Slabinja spring, Varoški bunar spring), which forms the border between these 2 countries.

According to the current findings, *Chaetopteryx bucari* is not rare in the Croatian fauna. In fact, it is one of the most dominant caddisflies in the Banovina region. Along with *Chaetopteryx fusca* (Kučinić 2002, Semnički et al. 2011, Cerjanec 2012, M. Kučinić unpublished data) it is one of the most frequently found species from genus *Chaetopteryx* in Croatia. *Chaetopteryx bucari* inhabits springs and headwaters of small streams. The only known larger limnocrene spring that *Chaetopteryx bucari* inhabits is the Pašino vrelo spring.

Taxa from the *Chaetopteryx rugulosa* group have allopatric distributions in Croatia (Figure 17): *Chaetopteryx bucari* is distributed in the Banovina region, *Chaetopteryx rugulosa rugulosa* in northern Croatia on Mt. Medvednica and Mt. Žumberak, *Chaetopteryx rugulosa mecsekensis* in eastern Croatia on Mt. Papuk and *Chaetopteryx marinkovicae* in the sub-Mediterranean part of Croatia in Istria (Malicky and Krušnik 1988, Malicky 1996, 2004, Oláh 2010). Systematic research in mountain areas in Lika and Gorski kotar (Kučinić 2002, Kučinić et al. 2008, Previšić and Popijač 2010, Cerjanec 2012, Semnički et al. 2011, 2012) and the Mediterranean part of Croatia (Dalmatia region) (Graf et al. 2008, Waringer et al. 2009, Vučković 2011, Vučković et al. 2011, M. Kučinić unpublished data) did not result in collections of *Chaetopteryx rugulosa* group species in these areas.

Many members of the genus *Chaetopteryx* are either small-scale endemics or species with a low number of disjunct populations. This makes the group very interesting for biogeographic studies. There are several reasons that could explain the observed pattern of distribution: small populations, poor mobility of the winter emerging adults, and distribution in springs and in headwater reaches of small streams. Besides naturally isolating individual populations from one another, these aspects can also cause difficulties for investigating the genus, as it is hard to access many of the sites, especially in winter. Future investigations of this genus will be focused on poorly researched areas in Croatia and the western Balkans to gain a better understanding of the distribution and biogeography of *Chaetopteryx* in the region.

## Supplementary Material

XML Treatment for Chaetopteryx bucari
